# Control programme for cystic echinococcosis in Uruguay

**DOI:** 10.1590/0074-02760160070

**Published:** 2016-06

**Authors:** Pilar Irabedra, Ciro Ferreira, Julio Sayes, Susana Elola, Miriam Rodríguez, Noelia Morel, Sebastian Segura, Estela dos Santos, Jorge A Guisantes

**Affiliations:** 1 National Commission for Zoonoses, Ministry of Public Health, Montevideo, Uruguay; 2 National Commission for Zoonoses, Ministry of Public Health, Tacuarembo, Uruguay; 3 National Commission for Zoonoses, Ministry of Public Health, Rivera, Uruguay; 4 University of Basque Country, Faculty of Pharmacy, Department of Immunology, Microbiology and Parasitology, Vitoria, Spain

**Keywords:** cystic echinococcosis, hydatidosis, hydatid disease, Echinococcus granulosus, zoonoses, parasitic diseases, Uruguay

## Abstract

Cystic echinococcosis is a highly endemic parasitic zoonosis that is present in the Southern Cone countries of America. For several decades, various prevention and control programmes have been implemented in different countries and regions, with varying results. In Uruguay, a new control programme was implemented in 2006 that employed new strategies for canine diagnosis and treatment, dog population control, diagnosis in humans, epidemiological surveillance, and health education, including community participation. The control programme in Uruguay addresses the control and surveillance of the disease from a holistic perspective based on Primary Health Care, which has strengthened the community’s participation in developing and coordinating activities in an interdisciplinary manner. Similarly, the control programme that is currently implemented is based on a risk-focused approach. The surveillance and control measures were focused on small villages and extremely poor urban areas. In this study, the strategies used and the results obtained from 2008-2013 are analysed and discussed.

Cystic echinococcosis (CE) is a parasitic zoonosis resulting from *Echinococcus granulosus* s.l., a tapeworm that belongs to the class Cestoda, family Taeniidae. The completion of the life cycle of these organisms requires two mammalian hosts: (i) a definitive host, such as dogs and other canids, in which the adult or strobilar phase develops in the small intestine, and (ii) an intermediate host, in which the cystic metacestode stage develops in different organs. The intermediate hosts can be one of a great number of herbivores, including cattle, sheep and goats, as well as other suitable hosts, such as pigs, horses and camels. Man might act as an aberrant intermediate host because the parasite cannot complete development in humans under natural conditions.

In endemic countries, CE has an important economic impact on human health and livestock production (Torgerson 2003). Today, CE is included in the group of neglected diseases (WHO 2010).

Since 1863, many programmes and control measures have been employed in several countries and have had varying outcomes (Craig & Larrieu 2007, Guisantes 2014). Several of these methods have been employed in islands, such as Iceland, New Zealand, Tasmania, the Falkland Islands, Sardinia and Cyprus.

Uruguay has a geographic area of 176,215 square kilometres. This area has a mild subtropical climate, with an annual average temperature of 17ºC and an average annual rainfall of 1,250 mm. The population contained 3,286,314 inhabitants in 2011 [Instituto Nacional de Estadística (ine.gub.uy/documents/10181/38317/Uruguay+en+cifras+2012/pdf)]. Animal husbandry is a significant industry in Uruguay: according to the 2008 data, the country contained 11,913,000 cattle and 9,558,000 sheep (MGAP 2008). CE is endemic throughout the country and is primarily associated with the dog/sheep cycle.

Since December 2005, the National Commission for Zoonoses (NCZ) of the Ministry of Public Health has conducted preventive and control programmes profiling CE. This entity replaced the National Commission against Hydatidosis (NCH), which was founded in 1965. The NCZ performs broader activities than did the NCH, including the control of other zoonoses. The NCZ has an inter-agency structure that facilitates the coordination of decision-making and executes the work lines and approved programmes.

Because Uruguay is divided into 19 departments, each department has a Committee of NCZ. The committees facilitate (i) the implementation of approved initiatives on the regional level, based on the NCZ guidelines, and (ii) the assessment of the performance of the work plans. Thus, the Departmental Committees have offices, materials and technical and administrative staff for these purposes.

The studies in dogs performed in 2004-2005, which focused on the detection of coproantigens, revealed that 6.4% of all rural settlements contained parasitised dogs (Elola et al. 2009). Similarly, pilot studies conducted in 2006 and 2007 using ultrasonography revealed the prevalence levels of hydatid cysts to be 1-2% among inhabitants of risk areas (Candau et al. 2009). The examined risk areas were rural areas, small population centres in rural areas and areas of critical socio-economic context.

These results prompted a redesign of the control programme of CE, implementing new lines of action and strengthening the existing actions. The purpose of this study was to present the strategies used in the control of hydatidosis in Uruguay and discuss the results obtained from 2008-2013.

## MATERIALS AND METHODS

One of the improvements of the current programme is its renewed focus on the actions taken in risk areas. These risk areas were identified by using the existing data on canine echinococcosis, parasitised livestock data from slaughterhouses, the number of cases of CE recorded in humans and the socio-environmental diagnosis of the analysed area (such as potable water supply, animal slaughter and offal disposal practices, predominance of sheep farming and socioeconomic and cultural status of the area).

The following actions have been developed as a part of the expansion of the control and surveillance programme:

*Launch of diagnosis in dogs* - The diagnosis of canine echinococcosis was performed using an ELISA test for *Echinococcus* coproantigen (CoproELISA) according to Morel et al. (2013). The dogs examined were obtained from scattered rural areas, small towns with risk characteristics and suburban areas of critical socio-economic context.

*Anthelminthic treatment of dog population* - The tapeworm treatment for dogs that were under control in rural areas was orally administered in the form of praziquantel (PZQ), every thirty days, at a dose of 5 mg/kg/bw. Since 2008, the treatment has also included broad-spectrum anthelmintics (pyrantel pamoate + PZQ + febantel) that were administered countrywide once a year to all registered dogs and up to three times per year in areas of critical socio-economic context, where the risk of other parasitic zoonoses, such as toxocariasis and ancylostomiasis, was detected through parasitological studies of dog faeces collected from the environment.

*Control of dog population* - In 2007, a voluntary and free surgical castration for owned dogs was introduced. Performed the spaying of dogs of both sexes. Veterinarians in mobile units throughout the country performed the spaying of dogs in both genres. These procedures were performed through the Working Days on Health (WDH) programme, which included health education and human diagnosis. Depending on the specific target population, only sterilisation sessions were conducted. This action was approved by the Society for the Protection of Animals of Uruguay and was performed along with a campaign on responsible dog ownership (Ferreira & Irabedra 2007, Guisantes 2009, Ferreira et al. 2011, Purpura 2011). In 2013, the practice of identifying sterilised dogs based on subcutaneously implanted microchips was introduced; these microchips included data identifying the dog owner.

The target population for the spaying programme was approximately 220,000 dogs, as estimated based on the number of people living in rural areas, towns with less than 5,000 inhabitants and slums. In Uruguay, the estimated ratio was 1 dog for every 2.9 inhabitants.

*Diagnosis in humans* - The diagnosis in humans was performed using ultrasonographic (US) surveys. The NCZ has 12 US mobile units, thereby facilitating access to remote places in the country. Physicians specialising in imaging technologies performed these ultrasound diagnoses. The US diagnoses in humans were voluntary. All persons studied were informed of the procedure and provided with brochures on the importance of the detection of asymptomatic carriers for early treatment. In Uruguay, there is a National Health Care System, and all positive patients are referred to an appropriate medical care in hospitals or health centres, where the treatment and monitoring of patients is ensured and a follow up is performed during medical or surgical treatment. The patients are also referred to a medical care centre when diseases other than cystic echinococcosis are detected.

*Health education* - Health education was conducted through the WDH at Public Health Centres. Verbal, visual and graphic methods were employed during this training. The US diagnosis also plays an important direct educational role, according to Kachani et al. (2003).

*Surveillance in Livestock* - The Ministry of Livestock, Agriculture and Fisheries supplied prevalence data on livestock, which were collected from slaughterhouse data. These data contributed to a trace-back analysis of the infected animals that arrived to abattoirs, thereby enabling the identification of infected farms and regions. There is an established protocol for researching both the epidemiological conditions surrounding the breeding of infected animals and the features of the farm of origin.

## RESULTS

**Fig. 1:**
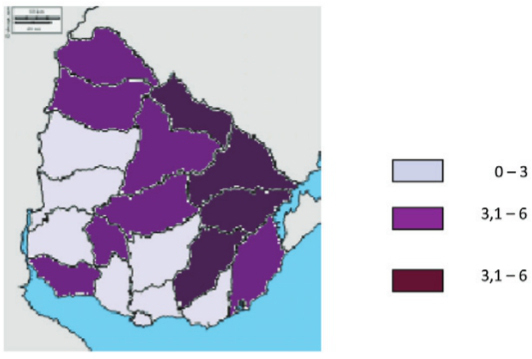
: results of the CoproELISA in the faeces of dogs analysed from 2008 to 2013, obtained in different Departments of Uruguay. The values are expressed as the percentage of positive dogs.

*Diagnosis in dogs* - Notably, CE is endemic throughout the country (with the exception of the Department of Montevideo), but there are differences according to the different regions. We considered risk areas to be those areas where dogs, livestock (especially sheep) and persons live together and where the dogs can easily access sheep or cattle offal due to the deficient conditions in slaughterhouses and the surrounding area. Many of these areas are of critical socio-economic context. For an area to be considered a CE risk area, in addition to the characteristics of livestock production and socio-demographic features, data on infected dogs are used. From 2008-2013, in addition to scattered rural areas, 376 small towns and suburban areas in 18 Departments (except the Department of Montevideo) have been studied for canine echinococcosis. According to the rates of canine parasitism from 2008-2013, the areas were classified into three ranges of risk: (i) areas with 0-3% parasitised dogs, which are considered low risk; (ii) areas with 3.1 to 6% infected dogs, which are considered medium risk; and (iii) areas with more than 6% infected dogs, which are considered high risk. The risk areas according to the rates of dog parasitism are shown in Fig. 1.

Between 2008-2013, the NCZ processed 7,013 faecal samples from dogs belonging to dispersed population centres and rural areas. In 2008, the NCZ initiated a study in small population centres with socio-demographic, economic and production characteristics of higher epidemiological risk. Until the implementation of this new programme, these areas had not been researched or analysed thoroughly. In 2011, we launched a programme focusing on the diagnosis of dogs, primarily in small towns and villages that had little access to health services. The results from 2008-2013 are shown in Table I.

**TABLE I:** Results of CoproELISA in dogs of towns and rural settlements from 2008-2011

Year	Small towns and suburban areas			Rural settlements (RS)		
	
	Number of towns	Number of dogs	Positive dogs (%)	Number of RS	Number of samples	Positive samples (%)
2008	25	731	72 (9.9%)	234	234	24 (10.2%)
2009	18	349	7 (2%)	202	202	9 (4.4%)
2010	12	258	12 (4.65%)	74	74	3 (4%)
2011	66	296	11 (3.71%)	ND	ND	ND
2012	74	484	32 (6.6%)	17* 84**	77 154	3 (3.9%) 17 (11.3%)
2013	181	3238	53 (1.6%)	615* 70**	909 145	15 (1.6%) 5 (3.4%)

Total	376	5356	187 (3.49%)	1296	1496* 299**	54 (3,6%)* 22 (7,35%)**

ND: no data; *rural settlements surveyed routinely; **rural settlements with parasitised livestock detected in slaughterhouses.

*Anthelminthic treatment of dog population* - The number of dogs dewormed with PZQ and broad-spectrum anthelmintics from 2008-2013 is shown in Table II.

**TABLE II:** Results of the anthelminthic treatment of dogs with praziquantel (PZQ) and broad-spectrum anthelmintics (BSA) from 2008-2013

	2008	2009	2010	2011	2012	2013	Mean
Number of dogs treated with PZQ*	116,560	113,000	109,000	108,089	103,254	98,921	103,138
Number of dogs treated with BSA	35,000	226,000	226,000	303,400	308,530	237,057	222,664

*average number of dogs treated with PZQ every 30 days.

*Control of dog population* - From 2008-2013, the NCZ performed 117,961 male and female castrations. In Montevideo, the capital of Uruguay, the procedures were conducted at 737 peripheral urban settlements or slums, where 31,973 dogs were spayed. These urban settlements have a human population of approximately 112,000 individuals, with an estimated dog population of 41,000 dogs. This number is based on a previous survey estimating the existence of one dog for every 2.96 inhabitants. The objective of the canine sterilisation programme was approximately 220,000 dogs, based on the estimated number of people living in risk areas (Fig. 1). Notably, Montevideo encompasses approximately half of the population of Uruguay, according to the Census of Population and Housing 2011 [Instituto Nacional de Estadística (ine.gub.uy/documents/10181/38317/Uruguay+en+cifras+2012/pdf)]. Canine surgical mortality is defined as death occurring within 10 days of surgery; thus, external causes, such as accidents or exposure to extreme temperatures, are not considered under this definition. The canine surgical mortality ratios in Montevideo were 0.22% in 2011, 0.14% in 2012 and 0.15% in 2013. Nationwide, 84% of the dogs that were spayed were females.

*Diagnosis in humans* - From 2008-2013, there were 87,536 US procedures performed in risk areas. The results are shown in Table III. In 2008, 2009 and 2011, no cysts were detected in people under 20 years of age. Among the 55 patients with hydatid cysts detected in 2010, only two individuals were under 20 years old. Among these two individuals, one patient was less than 10 years old. In 2012, only one patient was less than 10 years old, and in 2013, one patient was in the range of 11-20 years old.

**TABLE III:** Results of ultrasonographic surveys for human cystic echinococcosis (CE). Uruguay from 2008 to 2013

Year	Number of examined	CE +	Rate per 1,000 inhabitants
2008	23,763	150	6.5
2009	14,817	57	3.8
2010	17,184	55	3.2
2011	7,910	11	1.4
2012	12,425	21	1.7
2013	11,437	24	2.0

Total	87,536	318	3.6

*Health education* - From 2008-2013, the NCZ organised 561 WDH at the Public Health Centres distributed throughout the country. Educational reports and a practical guide to zoonoses were generated and delivered to all primary schools in the country. The NCZ also generated mass media advertising campaigns in newspapers, radio and television, focusing on the activities of the Commission.

*Surveillance in livestock* - The percentage of livers and lungs obtained from cattle and sheep confiscated in abattoirs with hydatid cysts are shown in Table IV.

**TABLE IV:** Livers and lungs confiscated in slaughterhouses for cystic echinococcosis in Uruguay in 2004, 2009 and 2013. Data from the Ministry of Livestock, Agriculture and Fisheries

Livestock		Percentage of confiscated viscera 2004	Percentage of confiscated viscera 2009	Percentage of confiscated viscera 2013
Cattle	Liver	11.0%	7.2%	5.35%
	Lungs	ND	5.7%	5.1%
Sheep	Liver	7.85%	5.5%	3.2%
	Lungs	ND	3.6%	2.9%

ND: no data.

## DISCUSSION

The control of CE is feasible according to the programmes conducted on islands such as Iceland, New Zealand, Falkland Islands or Tasmania, where the elimination of*E. granulosus* in dogs and livestock was attained, thus demonstrating that the eventual elimination of CE as a public health problem is possible (Craig & Larrieu 2007). The control of CE in these countries was achieved through island-based control programmes (Craig & Larrieu 2007). These programmes have been developed based on strategies that, in some cases, have some commonalities with the actions taken in countries of South America, such as mass deworming, laboratory testing, control of home slaughter, and veterinary inspection. However, the geographical conditions, types of livestock production and socioeconomic, cultural and anthropological characteristics are different.

The control programme implemented in Uruguay addresses the control and surveillance of the disease from a holistic view based on the Primary Health Care Centre, thereby strengthening the community’s participation in developing and coordinating activities in an interdisciplinary manner through WDH (Irabedra & Salvatella 2010, Ferreira et al. 2011). Similarly, the control programme that is currently implemented is based on a risk-focused approach (Ferreira & Irabedra 2007, Guisantes 2009). The surveillance and control measures were focused on small villages and extremely poor urban areas, where no targeted PZQ had been administered and where anti-helmintic treatment had been sporadic or irregular (Ferreira & Irabedra 2007).

During this study period (2008-2013), dog diagnosis via coproantigen detection using ELISA was introduced as a recommended technique due to its sensitivity and biosecurity for the technicians. The method has been used worldwide in many studies (Jenkins et al. 2000, Benito et al. 2006) and control programmes (Craig & Larrieu 2007, Ferreira & Irabedra 2007, Guisantes 2009, Ferreira et al. 2011). Currently, the following advantages of this technique have been described (Guisantes 2011): (i) positive results are obtained in the pre-patent period; (ii) this test an indicator of current infection; (iii) this test shows good sensitivity and specificity with some antigens and methods; (iv) this technique improves the results obtained using arecoline; (v) this technique can be applied to the analysis of individual cases or in larger studies; and (vi) a properly treated sample can be stored and shipped at room temperature. As shown in Table I, the CoproELISA results in dogs from small towns with risk characteristics and suburban areas of critical socio-economic context varied from 9.9% positive dogs in 2008 to 1.6% positive dogs in 2013, peaking in 2010 and 2012. In the case of the routine surveillance of dogs from rural settlements, the positive cases varied from 10.2% in 2008 to 1.6% in 2013. Notably, dogs from rural settlements surveyed routinely showed a mean of 3.6% positives, whereas dogs from rural settlements with parasitised livestock detected in slaughterhouses showed a positive mean of 7.35%. Fig. 1 shows that the areas of low risk correspond to most of the west coast and south of the country but that the areas of medium and high risk primarily correspond to areas in the centre, north, and east regions of the country (Fig. 1).

This study was conducted by tracing the infected animals that arrived at abattoirs and identifying their infected farms and regions. These results highlight the importance of tracking the infected livestock to identify areas where greater efforts in surveillance and control are needed. In this programme, tracing livestock by tracking the infected animals has facilitated the evaluation of the epidemiological and sanitary conditions in rural settlements of origin and the proposal of the appropriate corrective measures.

The anthelminthic treatment of the dog population shown in Table II, with a mean of 103,138 dogs treated with PZW every 30 days, revealed the good organisation of the fieldwork and an important coverage of dogs in the identified risk areas. Similarly, the broad-spectrum anthelmintics treatment is an important preventive action for other zoonotic helminthiases in dogs.

The current Control Programme of CE in Uruguay includes the voluntary and free surgical castration of owned dogs, which was initially introduced in 2007. The spaying of stray dogs is also currently performed. From 2008-2013, the NCZ performed 117,961 male and female castrations with a low post-operative mortality. These methods were approved by the Society for the Protection of Animals of Uruguay and were launched with a marketing campaign on responsible dog ownership (Purpura 2011).

US is used to assess the prevalence of CE in humans because this method is an excellent screening tool and is employed in several control programmes (Schantz 1997, Cohen et al. 1998, Larrieu et al. 2000, Frider et al. 2001, Wen et al. 2002, Macpherson et al 2003, Guisantes 2009). US is a technique with good sensitivity, specificity and clinical correlation. The use of this method in surveys has been highly accepted in the population (Kachani et al. 2003), and this low-cost method can be used to explore the abdominal organs and provide immediate results under field conditions (Gemmel et al. 2001). The results shown in Table III show that the number of detected cases of CE in humans has decreased from 6.5 per 1,000 inhabitants in 2008 to 2.8 in 2013. As previously discussed, a markedly low number of hydatid cysts was observed in children.

The surveillance surveys in CE must include not only data on human hydatidosis and canine echinococcosis but also data on CE in livestock (Schantz 1997, Guisantes 2014). These data are important for determining the prevalence of a zoonosis in a country or region prior to initiating and tracking the success of a control programme. The meat inspection in Uruguay that was performed in all slaughterhouses was officially authorised by the Ministry of Livestock, Agriculture and Fisheries. Veterinarians typically conduct this post-mortem inspection according to an established guideline. The data presented in Table IV are based on a high percentage of all animals slaughtered in this country. For example, in 2013, the data are based on 1,767,900 (87%) of the 2,009,300 cattle slaughtered in that year. For sheep, the data are based on 1,012,878 (61.3%) of the 1,651,191 sheep slaughtered. In Uruguay, the rate of infection in cattle decreased from 11% infected livers in 2004 to 5.35% infected livers in 2013. In sheep, the CE decreased from 7.85% to 3.2% during the same period. However, even if this reduction is important, the infection rates in livestock remain relatively high. Notably, Uruguay has a bovine herd of 11,913,000 animals and an ovine herd of 9,558,000 animals.

**Fig. 2:**
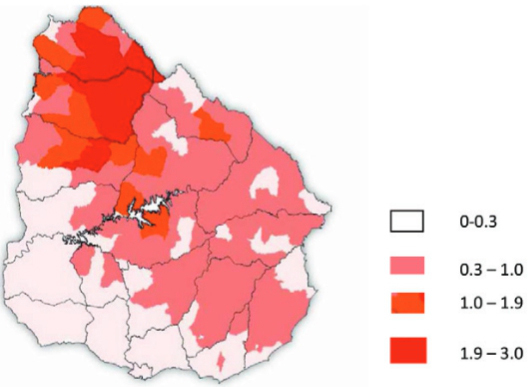
: number of sheep registered in different police sections of Uruguay (2010-2011), represented as a percentage over the total number of ovine livestock. The data were obtained from the Ministry of Livestock, Agriculture and Fisheries of Uruguay, Anuario Estadístico Agropecuario 2013.

As described above, in the control programme of the NCZ, the origin of the infected herds was tracked to evaluate the epidemiological and health conditions of the farms of origin. A comparison of the areas with the highest sheep flocks with the areas that have higher rates of infection in dogs revealed that the regions largely coincide (Figs 1-2).

The high community participation in the control programme was demonstrated as the number of WDH performed (1 WDH every 3.9 days on average). We considered health education to be one of the most important tools for the control and prevention of CE. Even in some control programmes, such as Iceland (1863-1960), WDH was the only action attempted for many years, and this technique recorded good results (Craig & Larrieu 2007). Health education is a multi-disciplinary activity that requires knowledge of medical sciences and of teaching and communication methods. According to Parodi et al. (2001), health education includes three types of activities that depend on one another: (i) information involves the transfer of expert knowledge to the targeted group. This activity highlights certain points to enable the community to actively participate in preventive actions. (ii) Health education *sensu strictu* targets groups that did not professionally concern the problem, such as school children or the public at large. (iii) Occupational training targets individuals who must implement health standards in professional activities (e.g., farmers and butchers). These activities were performed by the NCZ.

In addition to the activities described above, members of the NCZ have participated in and organised national and international events. These activities included a number of cooperative projects with control programmes for CE in other countries in South America.

As a sign of openness to the university and to assist in the training of future professionals with better knowledge on the control of zoonoses, a programme of internships in NCZ for students of the Faculty of Veterinary Medicine has been initiated in agreement with the state University. Notably, a control programme for management quality has been initiated. Based on the research and studies performed in Uruguay and other countries, the fight against hydatidosis will require a long-term strategy and solution. Thus far, the on-going strategy implemented through the NCZ has been successful, suggesting that Uruguay is on the right track to control CE.
